# Performance of novice versus experienced surgeons for dental implant placement with freehand, static guided and dynamic navigation approaches

**DOI:** 10.1038/s41598-023-29633-6

**Published:** 2023-02-14

**Authors:** Xiaotong Wang, Sohaib Shujaat, Jan Meeus, Eman Shaheen, Paul Legrand, Pierre Lahoud, Maurício do Nascimento Gerhardt, Reinhilde Jacobs

**Affiliations:** 1 OMFS IMPATH Research Group, Department of Imaging and Pathology, KU Leuven, Leuven, Belgium; 2 Department of Oral and Maxillofacial Surgery, The First Affiliated Hospital of Harbin Medical University, Harbin, China; 3 King Abdullah International Medical Research Center, Department of Maxillofacial Surgery and Diagnostic Sciences, College of Dentistry, King Saud bin Abdulaziz University for Health Sciences, Ministry of National Guard Health Affairs, Riyadh, Kingdom of Saudi Arabia; 4 Department of Oral and Maxillofacial Surgery, University Hospitals Leuven, Leuven, Belgium; 5 School of Health Sciences, Faculty of Dentistry, Pontifical Catholic University of Rio Grande do Sul, Porto Alegre, Brazil; 6 Department of Dental Medicine, Karolinska Institutet, Huddinge, Sweden

**Keywords:** Health care, Medical research

## Abstract

Lack of evidence exists related to the investigation of the accuracy and efficacy of novice versus experienced practitioners for dental implant placement. Hence, the following in vitro study was conducted to assess the accuracy of implant positioning and self-efficacy of novice compared to experienced surgeons for placing implant using freehand (FH), pilot drill-based partial guidance (PPG) and dynamic navigation (DN) approaches. The findings revealed that DN significantly improved the angular accuracy of implant placement compared with FH (P < 0.001) and PPG approaches (P < 0.001). The time required with DN was significantly longer than FH and PPG (P < 0.001), however, it was similar for both novice and experienced practitioners. The surgeon’s self-confidence questionnaire suggested that novice practitioners scored higher with both guided approaches, whereas experienced practitioners achieved higher scoring with PPG and FH compared to DN. In conclusion, implant placement executed under the guidance of DN showed high accuracy irrespective of the practitioner’s experience. The application of DN could be regarded as a beneficial tool for novices who offered high confidence of using the navigation system with the same level of accuracy and surgical time as that of experienced practitioners.

## Introduction

Dental implant surgery is a widely accepted therapeutic option for partially and fully edentulous patients. An ideal three-dimensional (3D) implant positioning and angulation is a prerequisite to ensure its long-term stable esthetic and functional outcome and to facilitate a correct prosthetic phase^1^. In contrast, non-ideal implant positioning may cause collateral damage to the vital anatomical structures within the vicinity of the placed implant and lead to certain intra-operative complications, such as maxillary sinus and/or cortical perforation, inferior alveolar nerve injury and damage to adjacent teeth^2^. Furthermore, an imprecise positioning of the implant has also been known to cause peri-implant bone loss and peri-implantitis at follow-up^2^. Hence, it is necessary that a surgeon should have a high level of experience and sufficient 3D spatial awareness to avoid complications associated with non-ideal placement of dental implants^3^.

The wide adoption of cone-beam computed tomography (CBCT) coupled with computer-aided design and computer-aided manufacturing (CAD/CAM) in a dental practice has improved the implant placement accuracy compared to freehand (FH) approach and allowed delivery of predictable prosthetically-driven treatment outcomes^4^. The two main computer-assisted techniques include implant placement with either CBCT-generated static surgical guide (SG) or dynamic navigation (DN) system^5^. Implant placement by static surgical guide can be classified as fully or partially guided, where full guidance refers to the control of all steps from osteotomy till implant placement through a guide^6^. It may be beneficial in cases with irregular bone quality, where minor implant movement is associated with higher deviation^7^. On the contrary, partial guidance (pilot drill or half-guided approach) involves only the use of a pilot drill or the complete osteotomy before implant placement is guided, followed by free-handed manual drilling and implant placement. A fully-guided approach offers less implant deviation compared to its partial counterpart^8^. However, pilot drill based partial guidance (PPG) is still the most commonly employed applied technique in a dental practice owing to its simplistic nature, reduction in irrigation problems, allowing minor implant position adjustment if required and easier control of implant placement in patients with limited mouth opening^9,10^.

Unlike SG-based approaches, DN system allows real-time guidance by tracking the optical markers fixed to the hand-piece and patient. Thereby, making it possible to monitor the drills and implant to follow the planned position^7^. It offers the advantage of real-time computer-guided freehand approach, where the operator has more freedom to adjust the implant position with the possibility of flapless surgery, lower morbidity, and a predictable outcome in both normal and complex cases with limited access or poor visualization^11,12^.

Recent studies have demonstrated that both SG and DN provide comparable accuracy which is higher than a FH approach for training novice surgeons, dental implant education and guiding experienced surgeons for a safe and predictable outcome^6,10,13–16^. However, to our knowledge no study exists investigating the accuracy and efficacy of novice versus experienced practitioners by comparing FH, SG and DN approaches. Therefore, the following study was conducted to quantitatively assess the accuracy of implant positioning and to qualitatively investigate the performance and self-confidence of novice surgeons compared to experienced surgeons for placing implants using FH, PPG and DN approaches.

## Material and methods

### Model fabrication

The study protocol was approved by the Ethical Review Board of the University Hospitals Leuven, Belgium (reference number: S64493). All experiments were performed in accordance with relevant guidelines and regulations. Informed consent was obtained from all participants. Inclusion criteria were CBCT dataset of lower jaw with sufficient bone quality and quantity for 3D model fabrication and implant placement, and a partial edentulous jaw with missing bilateral 1st molars. Exclusion criteria involved presence of pathological conditions or artefacts in the lower jaw.

A total of 36 identical simulation models with bilaterally missing 1st molar (72 implant placement sites) were designed in Mimics software (version 22.0, Materialise NV, Leuven, Belgium) and printed with Objet Connex 350 printer (Stratasys, Eden Prairie, MN, USA) using an acrylic-based resin (VeroDent MED670, Stratasys, Eden Prairie, MN, USA)^17^.

### Operators

An in-vitro study was conducted to compare three surgical protocols, which consisted of FH, PPG and DN system (Navident, ClaroNav, Toronto, Ontario, Canada). Three experienced dental practitioners with over 5-years of experience in implant surgery and three novice dental practitioners with no experience in surgical implantology participated in the study. All participants were trained and calibrated beforehand. The training session for navigation was provided to all practitioners which consisted of theoretical knowledge and drilling simulation practice to establish minimal proficiency for implant placement (at least 10 osteotomies on simulation models) with the DN system. Both experienced and novice practitioners had no prior training of using DN systems. Furthermore, novice practitioners were also provided with a theoretical and surgical simulation training by an experienced operator for using both FH and PPG approaches to optimally place implants.

All operators were randomly assigned the task of implant placement by FH, PPG or DN approach, with random sequence generated using Excel. In order to minimize bias, there was a one-week washout period between each method. Out of the 36 printed models (72 implant placement sites), 6 models (12 implant placement sites) were allocated to each operator, where they placed implants on 2 models by each approach (2 implant placement sites per model = 4 implants per approach).

### Treatment planning

The planning was performed by importing CBCT and intraoral scanned (IOS) images to an implant planning software (Blue Sky Plan 4, Blue Sky Bio LLC, Grayslake, IL, USA), where the implants were placed virtually, and surgical guides were designed following consultation with a consultant implantologist. Both the implant planning and SG were exported in standard tessellation language (STL) format. Subsequently, the surgical guide was fabricated with Objet Connex 350 printer and surgical sleeves were adhesively fixed onto the guide. Later, the CBCT dataset, IOS image and STL of virtual implant planning were imported to the user-interface of the DN system where virtual implants oriented identical to the planned position.

### Surgical procedure

The surgical procedure was standardized beforehand and the drilling sequence was prepared with irrigation following the manufacturer’s protocol using customized experimental In-Hex implants (3.8 mm × 9 mm, Wego, China). Implants were inserted using a motor unit (OsseoSet, Nobel Biocare AB, Goteborg, Sweden) at a speed of 15 rpm and a maximum torque of 50 N.cm. The drilling order was as follows: 2.2-mm-round drill followed by 2-mm pilot drill, 3.3-mm form drill, and 3.8-mm final drill.

**Figure 1:**
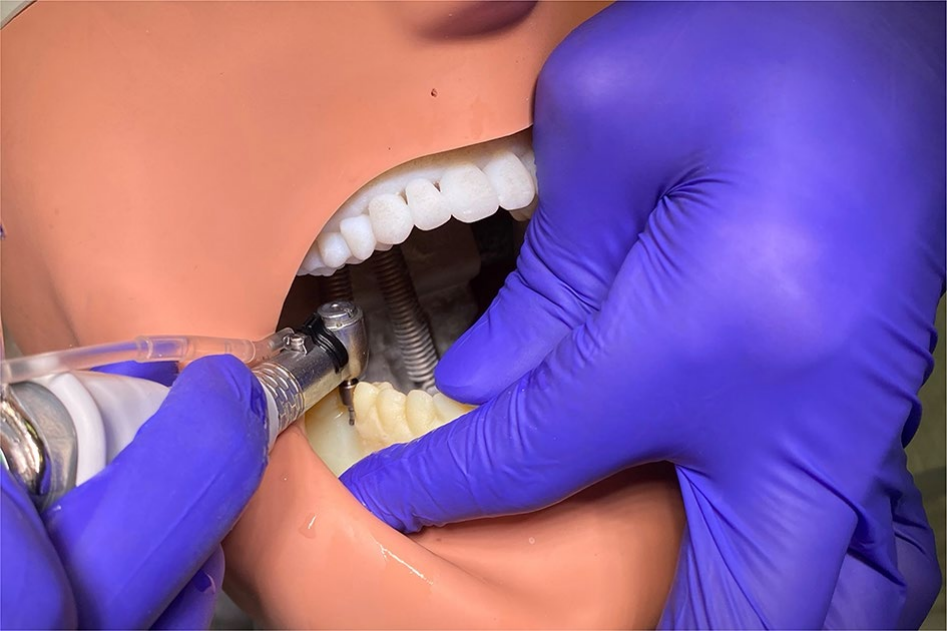
Freehand surgery.

**Figure 2:**
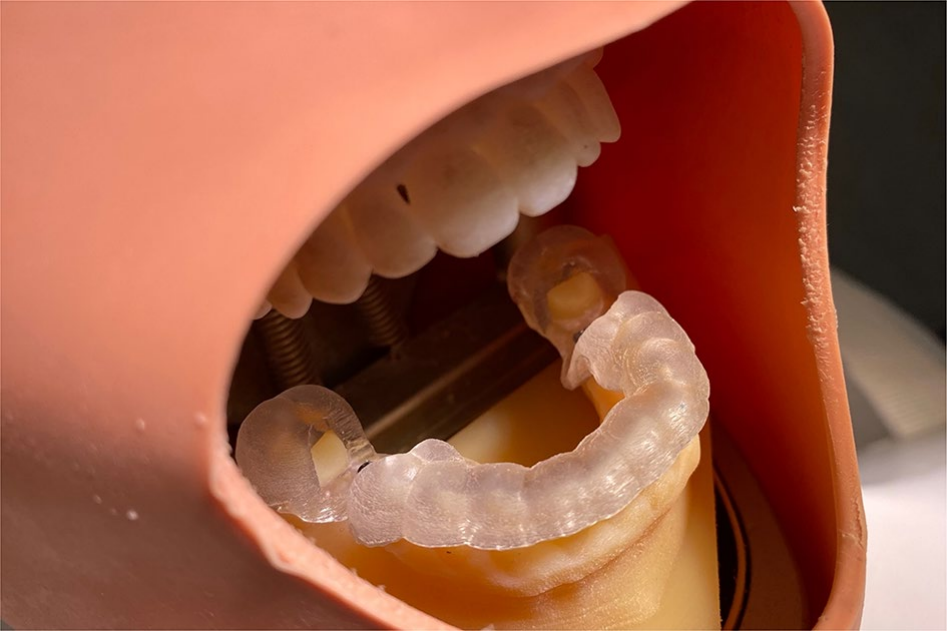
Surgical guide surgery. Surgical guide fitted intraorally.

Each printed model was placed in a dental phantom head to mimic a real clinical setting. During surgery with the FH approach, osteotomy drilling and implant placement were performed in accordance to the virtual surgical plan (Fig. 1). For the PPG, a tooth-supported guide was placed and a single pilot drill was used for the initial drill, followed by FH drilling (Fig. 2).

**Figure 3:**
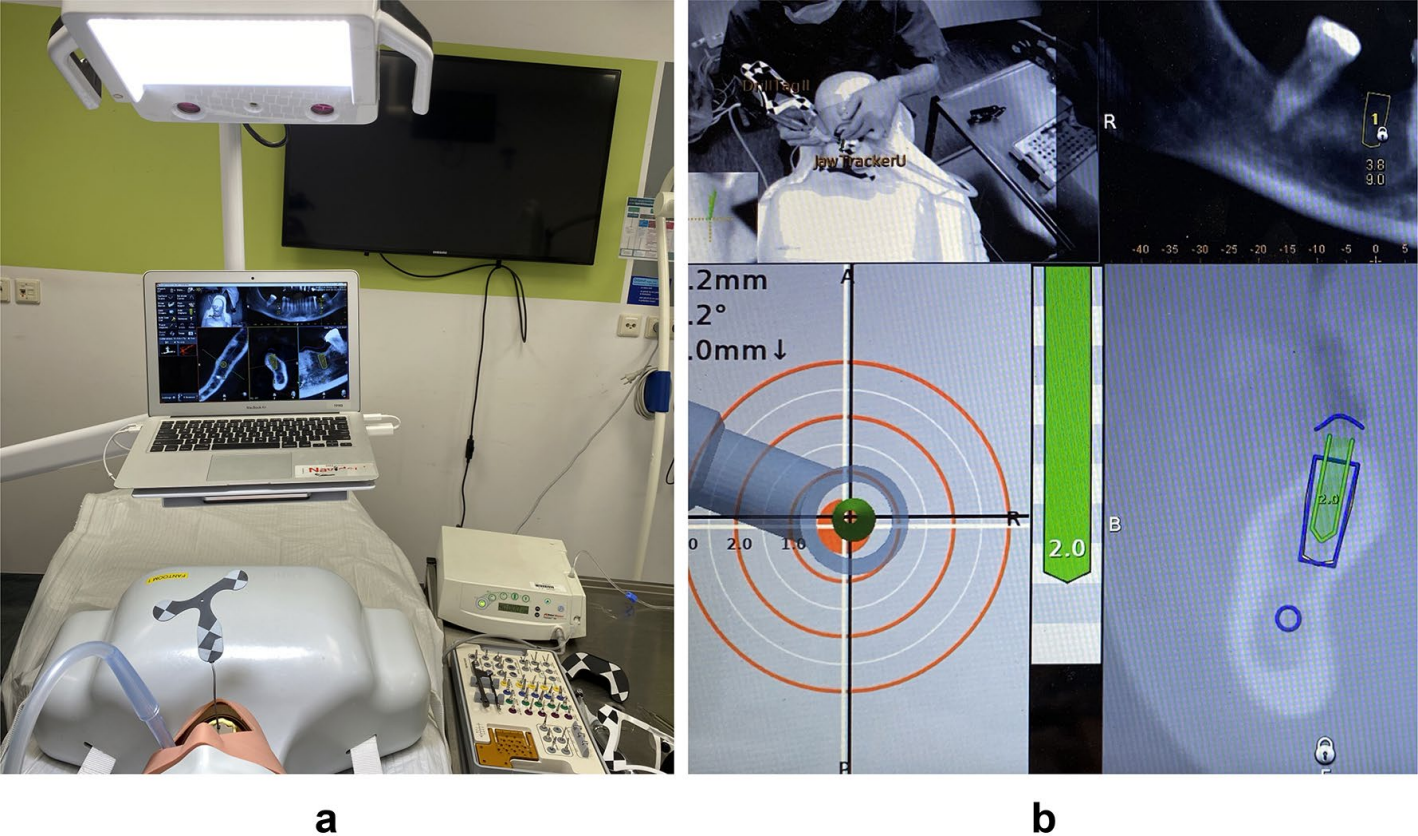
Navigation surgery. (**a**) Overview of navigation system; (**b**) Operation screen displays implant site preparation.

The DN approach involved firm attachment of the tracking tag to the anterior teeth of the jaw using silicone material (Fig. 3a). A tracking tag is a device which helps to maintain the registration between the jaw and its CBCT image and continuously tracks the patient’s jaw pose throughout the procedure. For guaranteeing a standard protocol, each operator used the same landmarks for the trace registration step. The registration accuracy was assessed to ensure optimal tracking prior to the surgical procedure. Each drill and implant required calibration before their insertion into the bone. The real-time visual feedback on a screen was used to guide the osteotomy preparation and the implant was placed according to the planned implant position. The location, angle and depth of drilling in relation to the predetermined treatment plan on the monitor were used to assist the practitioners (Fig. 3b).

### Performance evaluation

A validated self-confidence questionnaire was provided to all the practitioners, where they scored their performance with each approach on a combined scale of 1 to 30 (1 = least confident, 30 = most confident) based on six questions (Supplementary Table 1)^18^ This self-confidence scoring provided the “perceived self-efficacy” of the practitioners. In addition, time required for the procedure was also recorded.

### Accuracy assessment

**Figure 4:**
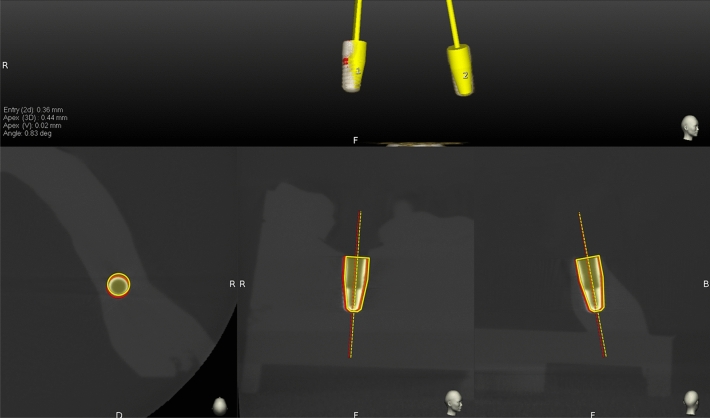
Superimposition of planned and actual placed implant positions.

Following implant placement, all models were scanned with a CBCT device (Accuitomo, J. Morita, Kyoto, Japan) using a standard CBCT scanning protocol (90 kV, 5 mA, 360° full-scan mode with Hi-Fi, 0.125 mm voxel size and 8 × 8 cm field of view). The pre‐operative CBCT scan with virtual implant position and the post‐operative CBCT scan with actual position were superimposed using EvaluNav software (ClaroNav Technology Inc., Toronto, Canada) as shown in Fig. 4. Thereafter, the planned and actual implant positions were compared automatically in the software by measuring the following variables: entry two-dimensional (2D) deviation (horizontal coronal deviation), apex 3D deviation (3D apical deviation), apex (V) deviation (vertical depth deviation) and angular deviation.

### Statistical analysis

Data were analyzed using IBM SPSS Statistics for Windows, version 21.0 (IBM Corp., Armonk, NY, USA). Descriptive statistics were calculated and presented as mean and standard deviation for entry 2D, apex 3D, apex V, angulation and surgical time. Self-confidence scores were examined by median and interquartile range. Normal distribution of residual values was assessed by means of normal quantile plots. The log-transformation was too strong, as confirmed by the points of the normal quantile plot which curved downward. Hence, a square-root transformation was applied for achieving normal distribution of the residual data. A linear mixed model with two crossed fixed factors (experience and method) and two random factors (surgeon and 3D printed model) were applied to evaluate the discrepancies amongst the three methods and the role of experience. Based on experience, as there were 3 novice and 3 experienced practitioners who inserted implants in two models, hence these were regarded as random factors. When the interaction was significant, a comparison was performed for methods per experience level and the experience level per method. Significance level was set at 5%.

### Ethics declarations

The study was approved by the Ethics Committee Research UZ/KU Leuven (reference number: S64493; registration number: B3222020000240).

## Results

Out of the total 72 implant placements, perforation of the lingual wall was observed at four implant sites (2 sites by experienced practitioners with FH approach; two sites by novice practitioners with PPG approach). Furthermore, a surgical guide was fractured by a novice practitioner during surgical drilling.

**Figure 5:**
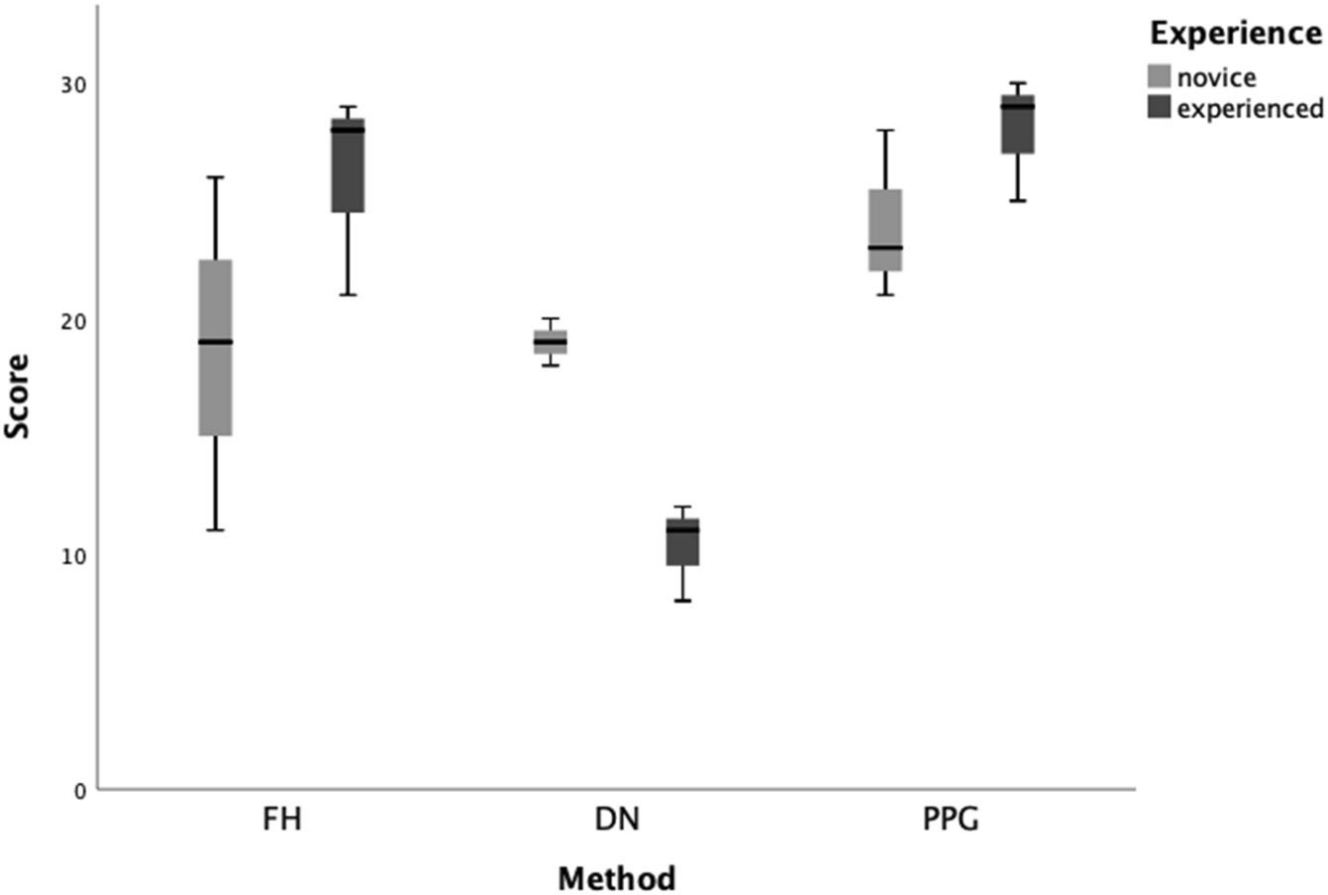
Median and inter-quartile range of the scoring of self-confidence assessment achieved with each method categorized by experience. Boxes comprise of 25th and 75th quartiles and median values, upper and lower whisker indicate highest and lowest values. *FH* freehand, *PPG* pilot drill based partial guidance, *DN* dynamic navigation.

According to the self-confidence questionnaire's scoring, overall navigation system scored significantly lower compared to freehand and surgical guided approaches (P = 0.007 and P < 0.001, respectively). Significant differences were observed based on the interaction of experience and approaches (P = 0.013). Experienced operators showed a high self-confidence score in favor of PPG, followed by FH and DN. However, overall novice practitioners perceived that their performance improved when using PPG and DN approaches as shown in Fig. 5.

Table 1 describes the mean deviation between planned and actual implant position and time consumption with each approach, where lower values indicate towards better implant positioning. The findings suggested that entry 2D deviation was lowest with the PPG approach, irrespective of operator’s experience. In addition, novice operators showed the highest apex 3D deviation using FH approach. The angular deviation was lowest with the DN, followed by PPG and FH approaches. Based on timing, implant placement using DN was most time-consuming and FH technique offered the least time consumption.

**Table 1:** Descriptive values (Mean ± SD) by surgery method and experience.

Method	Entry/mm	Apex (3D)/mm	Apex (V)/mm	Angle/°	Time/second
FH					
Experienced	1.11 ± 0.58	1.91 ± 1.06	0.54 ± 0.38	9.73 ± 4.29	196.25 ± 85.56
Novice	1.40 ± 1.01	2.54 ± 1.58	0.60 ± 0.33	8.15 ± 4.73	439.67 ± 203.97
PPG					
Experienced	0.83 ± 0.65	1.67 ± 0.94	0.48 ± 0.34	7.27 ± 3.82	217.25 ± 107.00
Novice	0.92 ± 0.38	1.66 ± 0.64	0.41 ± 0.27	7.07 ± 4.38	455.42 ± 130.34
DN					
Experienced	1.28 ± 0.55	1.70 ± 0.77	0.41 ± 0.25	4.03 ± 1.53	934.75 ± 773.24
Novice	1.07 ± 0.52	1.54 ± 0.94	0.70 ± 0.71	2.88 ± 2.51	900.58 ± 379.23

*FH* freehand, *PPG* pilot drill based partial guidance, *DN* dynamic navigation.

Based on linear mixed model analysis in Table 2, the angular deviation differed significantly amongst different surgical approaches (P < 0.001), where DN significantly improved the angular accuracy compared with FH (P < 0.001) and PPG approaches (P < 0.001). No significant differences were observed between experience of the operators (P = 0.10) or interaction of methods and experience (P = 0.57). In addition, the platform deviations (entry 2D, apex 3D and apex V) were similar irrespective of surgical approach, experience or interaction of both (P > 0.05).

**Table 2:** Statistical significance of implant deviation and time considering approach, experience, and interaction of both.

	Method	Experience	Method * Experience
Entry/mm	0.05	0.64	0.38
Apex(3d)/mm	0.07	0.59	0.31
Apex(v)/mm	0.47	0.45	0.61
Angle/°	** < 0.001**	0.10	0.57
Time/sec	** < 0.001**	** < 0.001**	**0.017**

Numbers in bold refer to statistically significant values.

The DN time was significantly longer (P < 0.001) compared with FH and PPG approaches (Table 2). A significant difference for interaction was observed (P = 0.017), which indicated that both experience and method affected the surgical time. Time required by experienced practitioners with the FH and PPG approaches was significantly faster than that of novice practitioners. However, the time required for DN was almost similar independent of the practitioner’s experience.

## Discussion

The present study evaluated the accuracy and performance of novice versus experienced practitioners for performing implant surgery using FH, PPG and DN approaches, which has not been thoroughly investigated in the prior available literature. The recorded parameters included accuracy of implant placement by assessing its deviation compared with the virtual plan, scores of self-confidence and operation time. The findings suggested that the overall navigation approach provided more accurate implant placement and offered equal time-consumption independent of the experience. Novice practitioners reported more confident with the DN approach compared to experienced practitioners. Furthermore, perforations in the lingual cortical region by FH and PPG were observed due to difficult access and the need for indirect visualization at the posterior region of the phantom. This issue did not exist with the DN approach as the practitioners could track the real-time drilling and implant positioning on a screen. A novice practitioner also fractured a SG during drilling. As the short inter-arch distance at the posterior region makes it difficult to appropriately place the drill in the sleeve of the surgical guide if a surgeon is not optimally trained, hence causing the guide to fracture.

Although the PPG offers the advantage of reducing the implant deviation in comparison with the FH method, there is still a risk of inaccurate implant positioning due to the difficulty of inserting the drills through the sleeves of the guide and keeping them in a centric position. The deviation for surgical guide might result from the tolerance between the sleeve and drill in surgical guide and the steps of digital workflow from data acquisition, software processing and template manufacturing^19^. In contrast, implant placements executed under the guidance of the DN system showed better accuracy, which was consistent with previous studies^20,21^. However, the cumulative error of navigation system might generate from the workflow of image processing, planning calibration, registration and surgical proficiency^16^.

The time required for FH and PPG was significantly faster than the DN approach. This was consistent with previous results that DN increased surgical time compared with FH^15,22^. The difference was due to the involvement of the necessary calibration steps for navigation throughout the surgical procedure. Another reasoning could be related to the competency of the technique related to hand–eye coordination, where the frequent use of navigation and mastering the approach might lower the surgical time. In contrast, the implant position with PPG, is completely dictated by the guide for the pilot drill and therefore the operators can quickly perform the drilling through the guide without strict monitor. Although DN requires a relatively longer surgical time compared to the FH or PPG, the potential time-efficiency in relation to planning or changing the surgical plan and delivery of the treatment on the same visit cannot be denied. In contrast, SG planning and manufacturing requires more time, especially in cases where third party companies are given the responsibility of guide preparation^11^. The surgical timing was significantly lower for experienced practitioners with FH and PPG, which could be due to their higher surgical proficiency. At the same instance, novice practitioners took longer as they required more time to orient and angulate drills. However, the DN approach led to almost similar timing irrespective of the experience.

The novice practitioners showed increased satisfaction with the assistance of guided approaches, which was consistent with previous studies^15,23^. This implies that novice practitioners tend to adapt to the guided technologies that help decrease the fear of surgical complications by conventional FH protocol. Furthermore, a high self-evaluation scoring was also confirmed by the improved accuracy.

Amongst the guided approaches, PPG received higher self-confidence scoring compared to DN by both novice and experienced practitioners. Due to the guidance by pilot-drill orientation, the later FH drilling offers more self-control to the practitioner, where a surgeon’s personal fine motor control allows determination of the correct implant positioning by manually inspecting each step. For navigation, the practitioners could self-control the motor depend on the guidance displayed by the tracking system. The low confidence reported in the application of navigation could be explained by that it requires a certain level of technical skills, manual dexterity and hand–eye coordination to perform the surgery while looking at the screen and avoiding any visual blockage of the tracking path. In spite of that, the novice practitioners believed that their performance improved with the navigation approach during the short training time compared with FH, which enables visualization of the osteotomy in real-time with minimal stress or risk of complications. On the other hand, experienced operators scored their self-satisfaction with DN even lower than novices because the experienced surgeons prefer the approach with well-documented higher success rate and are less prone to change by challenging an innovative treatment modality into practice^24^. However, it should be kept in mind that the ability of dynamic navigation to permit correction of implant positioning by displaying immediate feedback of the actual versus planned positioning of the drills and implant reduces the risk of harming patients compared to other approaches^14^.

The study had certain limitations. Firstly, the study was only limited to implant surgery at the site of lower mandibular 1st molar with small sample size, further studies should expand sample size and include other sites in both maxilla and mandible to assess its accuracy and performance of operators. Secondly, the simulated model lacked soft tissue and the factor of flap elevation was not assessed. Thirdly, the findings of this in vitro study should be interpreted with caution which might not be applied to patients in a real clinical setting. However, the applied approach could still act as an in vitro teaching model for improving novice surgeons’ dexterity and their skills before they perform the procedure on real patients. Finally, a PPG protocol was applied in the current study which is a more commonly used approach in a dental practice. However, future studies should also investigate the impact of half-guided and fully-guided approaches to reach a better conclusion.

## Conclusion

The dynamic navigation assisted implant placement technique significantly improved the angular accuracy of the implant placement compared with both FH and PPG approaches irrespective of the practitioner’s experience. The application of DN could be regarded as a more beneficial approach for novices who were more confident of using the navigation system for implant placement and were able to perform the procedure at the same level of accuracy and time as that of experienced practitioners.

## Supplementary Information

Supplementary Table 1.

## Data Availability

The datasets used and/or analysed during the current study are available from the corresponding author on reasonable request.
