# Supplementary material for: Performance of novice versus experienced surgeons for dental implant placement with freehand, static guided and dynamic navigation approaches
Source: Sci Rep. 2023 Feb 14;13:2598. doi: 10.1038/s41598-023-29633-6 (PMC9929278; doi:10.1038/s41598-023-29633-6)
Supplement: Supplementary file 1 — Supplementary Table 1. [file 41598_2023_29633_MOESM1_ESM.pdf]

Title: Performance of novice versus experienced surgeons for dental implant placement with freehand, static guided and dynamic navigation approaches

Xiaotong Wang[1], Sohaib Shujaat[2], Jan Meeus[3], Eman Shaheen[4] , Paul Legrand[5], Pierre Lahoud[6], Maurício do Nascimento Gerhardt[7], *Reinhilde Jacobs[8]

1. OMFS IMPATH Research Group, Department of Imaging and Pathology, KU Leuven, Kapucijnenvoer 33, 3000, Leuven, Belgium & Department of Oral and Maxillofacial Surgery, The First Affiliated Hospital of Harbin Medical University, Youzheng Street 23, Nangang, 150001, Harbin, China.

   xiaotong.wang@kuleuven.be
2. OMFS IMPATH Research Group, Department of Imaging and Pathology, KU Leuven, Kapucijnenvoer 33, 3000, Leuven, Belgium

   sohaib.shujaat941@gmail.com
3. Department of Oral and Maxillofacial Surgery, University Hospitals Leuven, Kapucijnenvoer 33, 3000, Leuven, Belgium

   jan.meeus@uzleuven.be
4. OMFS IMPATH Research Group, Department of Imaging and Pathology, KU Leuven, Kapucijnenvoer 33, 3000, Leuven, Belgium & Department of Oral and Maxillofacial Surgery, University Hospitals Leuven, Kapucijnenvoer 33, 3000, Leuven, Belgium

   eman.shaheen@uzleuven.be
5. OMFS IMPATH Research Group, Department of Imaging and Pathology, KU Leuven, Kapucijnenvoer 33, 3000, Leuven, Belgium

   legrand@telenet.be
6. OMFS IMPATH Research Group, Department of Imaging and Pathology, KU Leuven, Kapucijnenvoer 33, 3000, Leuven, Belgium

pierre.lahoud@kuleuven.be

7. OMFS IMPATH Research Group, Department of Imaging and Pathology, KU Leuven, Kapucijnenvoer 33, 3000, Leuven, Belgium & School of Health Sciences, Faculty of Dentistry, Pontifical Catholic University of Rio Grande do Sul, Porto Alegre, 90619-900, Brazil.

   mauricio_gerhardt@hotmail.com

8. Department of Dental Medicine, Karolinska Institutet, Solnavägen 1, 171 77 stockholm, Sweden & OMFS IMPATH Research Group, Department of Imaging and Pathology, KU Leuven, Kapucijnenvoer 33, 3000, Leuven, Belgium & Department of Oral and Maxillofacial Surgery, University Hospitals Leuven, Kapucijnenvoer 33, 3000, Leuven, Belgium

   reinhilde.jacobs@ki.se

Supplementary Table 1. Questionnaire for evaluating practitioner's self-confidence.

Please rate your performance on the procedure you just performed:

1. How confident were you during the procedure? **Total Score: /30**

1	2	3	4	5
Not at all		Confident		Very confident, level of attending surgeon

2. What was your surgical skill level during the procedure?

1	2	3	4	5
I felt I had none whatsoever		Average, as required of a resident my level		Well above average and only rarely encountered in a resident of my level

3. Were you worried during the procedure?

1	2	3	4	5
Constantly worried something was going to go wrong (life		Occasionally worried		I felt completely

threatening complication) or I was not operating at the required level				calm, not worried at all

4. Were you anxious during the procedure?

1	2	3	4	5
Constantly anxious, I had "flutters in my stomach" during the whole procedure		Occasionally anxious		Not anxious at all

5. Based on your performance today, would you have liked to have avoided this procedure altogether?

1	2	3	4	5
Indeed		Only occasionally during the procedure		On the contrary, I would do it again anytime

6. How comfortable were you with the independent planning and performing of the procedure?

1	2	3	4	5
Very uncomfortable		Average		Very comfortable (level of attending surgeon)
